# Nitric Oxide and Brazilian Propolis Combined Accelerates Tissue Repair by Modulating Cell Migration, Cytokine Production and Collagen Deposition in Experimental Leishmaniasis

**DOI:** 10.1371/journal.pone.0125101

**Published:** 2015-05-14

**Authors:** Milena Menegazzo Miranda, Carolina Panis, Allan Henrique Depieri Cataneo, Suelen Santos da Silva, Natalia Yoshie Kawakami, Luiz Gonzaga de França Lopes, Alexandre Tadachi Morey, Lucy Megumi Yamauchi, Célia Guadalupe Tardelli de Jesus Andrade, Rubens Cecchini, Jean Jerley Nogueira da Silva, José Maurício Sforcin, Ivete Conchon-Costa, Wander Rogério Pavanelli

**Affiliations:** 1 Department of Pathology Science, Center of Biological Sciences, State University of Londrina, Londrina, Paraná, Brazil; 2 Laboratory of Inflammatory Mediators, State University of Western Paraná, Francisco Beltrão, Paraná, Brazil; 3 Department of Organic and Inorganic Chemistry, Federal University of Ceará, Fortaleza, Ceará, Brazil; 4 Department of Microbiology, Center of Biological Sciences, State University of Londrina, Londrina, Paraná, Brazil; 5 Laboratory of Electron Microscopy and Microanalysis, Department of General Biology, Center of Biological Sciences, State University of Londrina, Londrina, Paraná, Brazil; 6 Department of Chemistry, State University of Roraima, Boa Vista, Roraima, Brazil; 7 Department of Microbiology and Immunology, Biosciences Institute, São Paulo State University, Botucatu, São Paulo, Brazil; Pasteur Institute of Iran, ISLAMIC REPUBLIC OF IRAN

## Abstract

The fact that drugs currently used in the treatment of *Leishmania* are highly toxic and associated with acquired resistance has promoted the search for new therapies for treating American tegumentary leishmaniasis (ATL). In this study, BALB/c mice were injected in the hind paw with *Leishmania (Leishmania) amazonensis* and subsequently treated with a combination of nitric oxide (NO) donor (cis-[Ru(bpy) _2_imN(NO)](PF_6_)_3_) (Ru-NO), given by intraperitoneal injection, and oral Brazilian propolis for 30 days. Ru-NO reached the center of the lesion and increased the NO level in the injured hind paw without lesion exacerbation. Histological and immunological parameters of chronic inflammation showed that this combined treatment increased the efficacy of macrophages, determined by the decrease in the number of parasitized cells, leading to reduced expression of proinflammatory and tissue damage markers. In addition, these drugs in combination fostered wound healing, enhanced the number of fibroblasts, pro-healing cytokines and induced collagen synthesis at the lesion site. Overall, our findings suggest that the combination of the NO donor Ru-NO and Brazilian propolis alleviates experimental ATL lesions, highlighting a new therapeutic option that can be considered for further *in vivo* investigations as a candidate for the treatment of cutaneous leishmaniasis.

## Introduction

*Leishmania (Leishmania) amazonensis* is an obligatory intracellular parasite of mammalian cells and one of the causative agents of American tegumentary leishmaniasis (ATL). Infection occurs from the bite of an infected phlebotomine sand fly, and depending on the host immune response, various clinical manifestations may result, ranging from a single granulomatous skin lesion at the site of the bite to diffuse lesions, where it may or may not affect the mucous membranes or even progress to visceral disease [1].

The first choice of antiparasitic agent for treating this disease in Brazil since the 1940s has been Glucantime (N-methyl glucamine antimoniate); however, despite being used against all forms of leishmaniasis, it has serious limitations in clinical practice, due to its extensive toxicity and limited efficacy [2].

Experimental models have shown that the outcome of *Leishmania* infection is critically dependent on the activation of CD4 T cell subsets [3]. Host susceptibility is ascribed to Th2 immune response, which leads to the development and multiplication of the parasite [4]. On the other hand, resistance is established by preferential activation of a Th1 subpopulation of lymphocytes, which produce IFN-γ and TNF-α, leading to macrophage activation and increased activity of inducible nitric oxide synthase (iNOS) and NADPH oxidase, with consequent increase in the production of nitric oxide (NO) and reactive oxygen species (ROS), respectively [4–6]. Among the microbicidal mechanisms exhibited by phagocytic cells, NO production has been shown to be one of the most important for eliminating *Leishmania* spp.[7, 8].

Nevertheless, *Leishmania* has numerous escape mechanisms [9]. For instance, it can reduce NO production in macrophages by increasing the expression of arginase, which catalyzed the cleavage of L-arginine [10–12]. Therefore, the parasite can multiply and escape from the microbicidal actions of the host, and in attempt to eliminate it and control the infection, there is an intense activation of many defense mechanisms, resulting in a strong inflammatory response, increasing tissue damage and exacerbation of the injury [13, 14].

Synthetic or natural substances with leishmanicidal and even anti-inflammatory capacity have emerged as an alternative to conventional treatment. In this context, the use of exogenous compounds that can donate microbicidal molecules, such as NO, can be an important strategy for the effective treatment of leishmaniasis. Some *in vitro* studies had already reported that NO donors can inhibit mitochondrial respiration in amastigote and promastigote forms of *Leishmania* spp., killing the parasite [15, 16]. Moreover, NO plays an important role in wound healing and collagen deposition [17, 18]. Accordingly, ruthenium-NO-donor complexes have emerged as a potential therapeutic approach for disease models that require NO as the effector microbicidal molecule. The advantages of such drugs include controlled release of NO, low toxicity and stability in aqueous media. NO molecules are released from such complexes by reducing agents commonly present in biological media [19, 20].

Another compound of interest that has been widely investigated in parasitic infections is propolis extract. Propolis has been widely employed in several disease models, showing great potential in protective immune response against leishmaniasis [21–23]. The major components of this bee product are phenolic compounds (flavonoids, aromatic acids and benzopyranes), di- and triterpenes and essential oils [24], which have already been described as having antiinflammatory [25, 26], antioxidant [27], immunomodulatory [28–31] properties and being able to promote wound healing and reepithelization process [32, 33]. Furthermore, it has been shown that propolis is able to increase the generation of hydrogen peroxide, suggesting that this product modulates the activation of macrophages and acts against intracellular parasites [34, 35].

Since NO and propolis can play an important role in the control of various parasite diseases, the objective of this study was to evaluate the effect of combined therapy using the NO donor cis-[Ru(bpy)_2_imN(NO)](PF_6_)_3_ (Ru-NO), where bpy = 2,2’-bipyridine and imN = imidazole, and Brazilian propolis against infection with *L*. *amazonensis*.

## Material and Methods

### Parasite

*L*. *amazonensis* (MHOM/BR/1989/166MJO) was used as promastigote forms in the stationary growth phase for the infection. The parasites were obtained from popliteal lymph nodes of *L*. *amazonensis*-infected mice and maintained in 199 culture medium (GIBCO-Invitrogen) supplemented with 10% fetal bovine serum (FBS; GIBCO-Invitrogen), 1 M Hepes, 0.1% human urine, 0.1% L-glutamine, 10 U/mL penicillin and 10 μg/mL streptomycin (GIBCO-Invitrogen) and 10% sodium bicarbonate. Cells were maintained in 25-cm^2^ flasks in a BOD-type incubator at 25°C.

### Chemicals, drugs, and reagents

Propolis was collected in the Beekeeping Section of the Lageado Farm, São Paulo State University (UNESP), Botucatu, SP, Brazil, from *Apis mellifera* L. colonies. Propolis was ground and a 30% extract in 70% ethanol was prepared. Its chemical composition was analyzed using gas-chromatography (GC), gas chromatography-mass spectrometry (GC-MS) and thin layer chromatography (TLC) [24]. The final concentration of the solvent, ethanol, in the experiments did not exceed 0.1%. NO donor compound cis-[Ru(bpy)_2_imN(NO)](PF_6_)_3_ (Ru-NO) was synthesized and characterized as described previously by Silva *et al*. [36]. Glucantime (Sanofi-Aventis, Brazil; 300 mg/ml, 81 mg/ml Sb^V^) was used as standard drug for treatment of ACL[15].

### Animals and infection

Female BALB/c mice weighing approximately 25–30 g, aged 6–8 weeks old, were obtained from the Institute Carlos Chagas-Fiocruz, Curitiba-PR, Brazil. They were infected in the right hind paw with 1x10^5^ *L*. *amazonensis* promastigote forms. Mice were kept under pathogen-free conditions and used according to protocols approved by the Institutional Animal Care and Use Committee. This study was approved by Londrina State University Ethics Committee for Animal Experimentation (No. 56/2012).

### Experimental procedures

Groups of five *L*. *amazonensis-* infected BALB/c were treated for 30 consecutive days, starting after lesion appearance in all mice, which occurred 8 weeks post-inoculation (p.i.). The treatment groups were as follows: Glucantime (33 μmol.kg^-1^ day^-1^) by intraperitoneal (i.p.) injection; propolis diluted in PBS, administered orally (p.o.) (5 mg.kg^-1^ day^-1^) [35]; Ru-NO diluted in PBS (0.385 μmol.kg^-1^ day^-1^, i.p.) [37]; and Ru-NO (0.385 μmol.kg^-1^ day^-1^, i.p.) plus propolis (5 mg.kg^-1^ day^-1^, p.o.). Uninfected and infected mice were used as control groups and received only PBS vehicle (p.o. and i.p.). The lesion size (in mm) was measured weekly during the treatment using a digital caliper (Starrett 799). At the end of therapy (86 days p.i.), animals were euthanized. Plasma was collected and the injured hind paw of each animal was excised, which was divided into four fragments for analysis.

### AST and ALT levels

Five BALB/c mice were treated for 30 consecutive days with Ru-NO (0.385 μmol.kg^-1^ day^-1^, i.p.) plus propolis (5 mg.kg^-1^ day^-1^, p.o.). Plasma was collected for measurement of aspartate aminotransferase (AST) and alanine aminotransferase (ALT), markers of hepatocellular damage, using a colorimetric assay with a commercial kit from Labtest Diagnóstica (Lagoa Santa, MG, Brazil).

### Ruthenium detection by energy dispersive spectroscopy (EDS)

Injured paw fragments were immersed in 8% paraformaldehyde and 0.2 M cacodylate buffer fixing solution for 48 h at room temperature. The paws were then dehydrated in an increasing graded ethanol series (70, 80, 90 and 100% GL) and critical-point dried in CO_2_ (Bal-Tec CPD 030). The samples were placed on stubs and coated with carbon using a sputter coater (Bal-Tec SCD 050). For the detection of ruthenium, samples were analyzed by spectroscopy energy dispersive (EDS, Oxford), INCA software, coupled to a scanning electron microscope (SEM) FEI Quanta 200.

### Real-time detection of NO/peroxynitrite by high sensitivity chemiluminescence

NO production was evaluated employing a highly sensitive NO detection system described by Kikuchi et al. (1993) [38], with some modifications. In this method, NO/peroxinitrite reacts with hydrogen peroxide, which in the presence of luminol produces triplet oxygen, which decays to singlet oxygen and emits photons, detected by a luminometer system coupled to a computer. One of the hind paw fragments was mechanically homogenized (Tissue-tearor, BioSpec), and the supernatants (100 mg/ml) were diluted 1:1 in fresh sterile 2 mM Na_2_CO_3_ buffer, pH 8.5, previously degassed with bubbling N_2_ for 20 min to eliminate the presence of molecular oxygen and oxidation of NO to nitrite/nitrate. The final reaction volume was 500 μl of macerated paw plus 500 μl of Na_2_CO_3_ buffer. The starting reagent was prepared by mixing equal volumes of luminol solution (4.39 μM dissolved in 1 M KOH) diluted 1:10 in 36.58 μM desferrioxamine and 2.44 μM H_2_O_2_, with 3 parts degassed Na_2_CO_3_ buffer. This mixture was vortexed for 5 min before use. All solutions were sterile and kept at 25°C in covered tubes, protected from light. Finally, the luminometer chamber was injected with 50 μl of starting reagent and the reaction was performed in a Glomax luminometer (Promega), with automatic reagent injector, employing a kinetic protocol which allowed following the reaction at 10 readings per second. All chemicals were purchased from Sigma.

### DNA extraction and parasite quantification by real-time PCR

Real-time quantitative PCR (RT-qPCR) was performed to determine the tissue parasite load in each group. A hind paw fragment was weighed, washed in PBS, and homogenized in lysis buffer (50 mM Tris-HCl [pH 7.6], 10 mM EDTA, 0.5% SDS, and 0.2 mg/ml proteinase K (Invitrogen, Carlsbad, CA), followed by phenol-chloroform extraction of DNA. Briefly, samples were mechanically homogenized (Tissue-tearor, BioSpec), incubated at 55°C for 12 h, and extracted twice with phenol-chloroform-isoamyl alcohol (25:24:1). Two volumes of cold ethanol (Merck) were added to the aqueous phase, and samples were stored at -20°C for 12 h. Samples were then centrifuged for 30 min at 10,000 *g*, washed with 70% ethanol, dried at room temperature, and resuspended in 10 mM Tris HCl (pH 8.5) [39]. Real-time PCR was performed by using Platinum SYBR Green qPCR SuperMix UDG with ROX reagent (Invitrogen Corporation, New York, NY) with 100 ng total genomic DNA (gDNA). Parasite quantification was performed using JW11 (forward, 5′-CCTATTTTACACCAACCCCCAGT-3′) and JW12 (reverse, 5′-GGGTAGGGGCGTTCTGCGAAA-3′) *Leishmania*-specific primers [40]. The samples were amplified with a Corbett Rotor-Gene thermal cycler under the following PCR conditions: an initial step of 2 min at 50°C, a second step of 10 min at 95°C, and 40 cycles of 30 s at 95°C, 30 s at 57°C, 30 s at 72°C, and 15 s at 82°C, followed by a dissociation step. The results were based on a standard curve constructed with DNA from culture samples of *L*. *amazonensis* promastigote forms.

### Histological analysis

Hind paw fragments collected were fixed in Bouin’s solution, decalcified for 45 days in 5% EDTA, processed for paraffin embedding, sectioned (4 μm) and stained with hematoxylin-eosin (H&E) or sirius red by the picrosirius technique to assess the presence of collagen. Cellular profile was scored by counting the different cell types (macrophages, vacuolated macrophages, fibroblasts and lymphocytes) in H&E-stained paw sections, analyzed with a photomicroscope (Olympus, Miami, FL, USA) at a final magnification of 200x. Five images from each mouse were captured using Motic Images Plus v.2.0 (Motic China Group Co. Ltd., Xiamen, China). These images were divided into four quadrants, two of which were randomly selected for cell quantification with ImageJ 1.45 s software (NIH, USA—2011). Collagen quantification of the lesion site was determined in sirius red-stained paw sections under polarized light using a photomicroscope (Nikon Eclipse 80i) with a camera (Nikon DSFi1C) coupled to a computer using Nis Element software (Shinjuku, Japan), at a final magnification of 200x. Four images of four sections from each mouse were considered for the study and analyzed by Image Pro Plus (version 4.5). The results were expressed as percentage of area with the presence of collagen compared to the total measured area.

### Immunohistochemistry

The paw sections were also analyzed by immunohistochemistry [41] to identify CD4+ and CD8+ T lymphocytes, iNOS and nitrotyrosine (3NT) by the labeled streptavidin—biotin method using an LSAB kit (DAKO Japan, Kyoto, Japan). The paraffin-embedded sections were deparaffinized and rehydrated, treated for 40 min with 2% BSA and incubated overnight at 4°C with primary antibody (anti- CD4^+^, anti- CD8^+^, anti-iNOS and anti-3NT rabbit polyclonal antibody diluted 1:500, Sigma). Horseradish peroxidase activity was visualized by treatment with H_2_O_2_ and 3, 30—diaminobenzidine (DAB) for 5 min. In the last step, the sections were weakly counterstained with Harry’s hematoxylin (Merck). For each assay, negative controls were prepared on serial sections. Intensity and localization of immunoreactivity with all primary antibodies used were examined on all sections using a light microscope (Olympus BX41, Olympus Optical Co., Ltd., Tokyo, Japan). As a negative control, the primary antibody was omitted. For the image analysis study, color photomicrographs of representative areas (magnification of 400x) were digitally acquired. For semi-quantitative scoring, images were evaluated by using the color deconvolution tool in Image J software (NIH, USA). Pixels were categorized as previously described by Chatterjee et al. [42] as strong positive (3+), positive (2+), weak positive (1+) and negative (0).

### Determination of cytokine profile by Western blotting

The supernatants from hind paw fragments (100 mg/ml) was used to measure the cytokines and transcription factor expressed in the lesion by Western blotting [43]. Supernatant samples were first centrifuged and total protein determined by Lowry's method modified by Miller [44]. Supernatant samples were diluted 1:2000 in 0.9% NaCl and incubated with 300 μl of cupric reagent for 10 min. Afterwards, the mixture was vortexed and added to 900 μL of Folin-Ciocaulteau's reagent and kept at 50°C in a water bath for 10 min. Absorbance of the samples was read at 660 nm, and the protein concentration was obtained from the standard curve of bovine serum albumin. A volume of sample corresponding to 20 μg protein was subjected to 10% acrylamide gel electrophoresis followed by transfer to a nitrocellulose membrane [43]. Membranes were blocked in 5% milk and incubated overnight with antibodies to NF-κB (p65), STAT3, TNF-α, IL-10 and TGF-β1 diluted 1:1000 (Santa Cruz Biotechnology, USA). Subsequently, the membranes were washed in PBS-0.1% Tween for 30 min and incubated with secondary antibody specific for each primary antibody for 2 h at room temperature (diluted 1:2000). The detection of the bands of respective antibodies was performed using the ECL Plus kit (GE Healthcare, United Kingdom). The images of the bands were scanned (Labscan Software, EG Healthcare) and quantified in Image J software (NIH, USA).

### Statistical analysis

Results were expressed as the mean ± standard error of the mean (SEM) and analyzed with the Prism 5.0 statistical program (GraphPad Software, San Diego, CA), using the Student t-test, comparing with infected control. Differences were considered significant when *P* < 0.05.

## Results

### Ru-NO by intraperitoneal injection was able to reach the site of *Leishmania*-induced lesion

**Fig 1:**
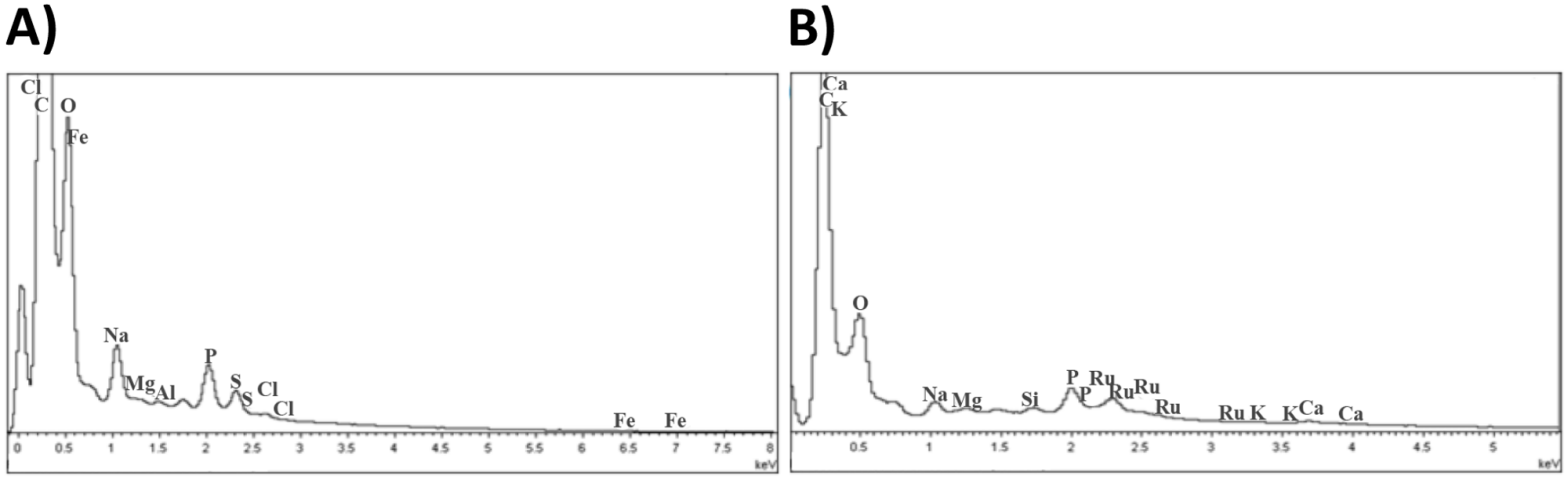
Ru-NO by intraperitoneal injection is able to reach the center of the lesion. Animals were inoculated with 1 x 10^5^ *L*. *amazonensis* promastigote forms in the right hind paw, and after lesion appearance, they were treated intraperitoneally with Ru-NO (0.385 μmol.kg^-1^ day^-1^). **A)** EDS analysis of paw sections was performed in infected control and **B)** RuNO treated group, to identify the elements present at the lesion site.

Initially, we checked if the route of drug administration delivered Ru-NO to the injured paw. Energy dispersive spectroscopy (EDS) analysis of the infected control revealed the presence of many elements including carbon, oxygen, iron, sodium, chlorine, phosphorus, magnesium and sulfur in the paw section (Fig 1A). Ru-NO treated mice showed the same elements plus the presence of ruthenium, proving that the intraperitoneal injection of Ru-NO donor was sufficient to ensure the delivery of this compound to the lesion site (Fig 1B). The presence of silicon and calcium are from mouse hair and bone, respectively.

### Intraperitoneal injection of Ru-NO increased NO levels at the lesion site

Experimental interest in the use of NO-donors in ATL is based on the fact that *Leishmania* parasites are able to deplete NO levels by several mechanisms, reducing the bioavailability of NO for microbicidal action [9].

The complex cis-[Ru(bpy)_2_imN(NO)](PF_6_)_3_ releases NO through electrochemical, photochemical and chemical processes [36]. The reaction of this compound with antioxidants could lead to the availability of free NO [37]. Since we demonstrated that the drug reached the lesion, we wondered if NO was released at the site of injury, employing a high-sensitivity chemiluminescence method based on the detection of NO/nitrosative stress [38].

**Fig 2:**
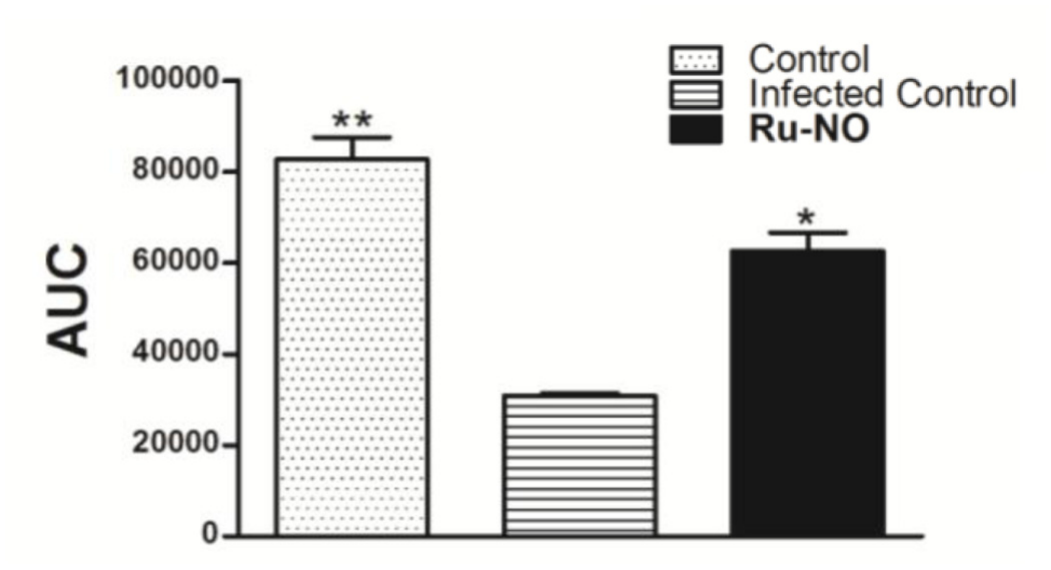
NO level was increased in the lesion after treatment with Ru-NO. NO/peroxynitrite was estimated by chemiluminescence in supernatant of macerated paw. The result was expressed as the mean ± SEM of five animals per group. Bars are represented by the medians of each group. (AUC: area under the curve). Significant difference relative to the infected control * *P*<0.05 and ** *P*<0.01, unpaired t-test.

According to our results, the infected group showed a marked depletion of NO levels when compared to uninfected control (Fig 2). Infected BALB/c mice treated with Ru-NO, i.p., showed increased NO levels at the lesion site, indicating its local release by the Ru-NO donor (Fig 2), which demonstrated that systemic administration of Ru-NO donor could represent a useful strategy for NO release at the lesion site, overcoming the NO depleting mechanisms of *Leishmania*.

### Treatments delayed the development of the cutaneous lesion and significantly reduced the parasite load

Several studies have reported that Brazilian propolis exerts important immunomodulatory effects [22, 23], which could improve the effects of the Ru-NO donor against the *Leishmania* pathogenic mechanisms. Therefore, we aimed to investigate the immunomodulatory effect of combined propolis and Ru-NO in experimental leishmaniasis. BALB/c mice were infected with *L*. *amazonensis* and the lesion was monitored for 30 days, with or without treatments.

**Fig 3:**
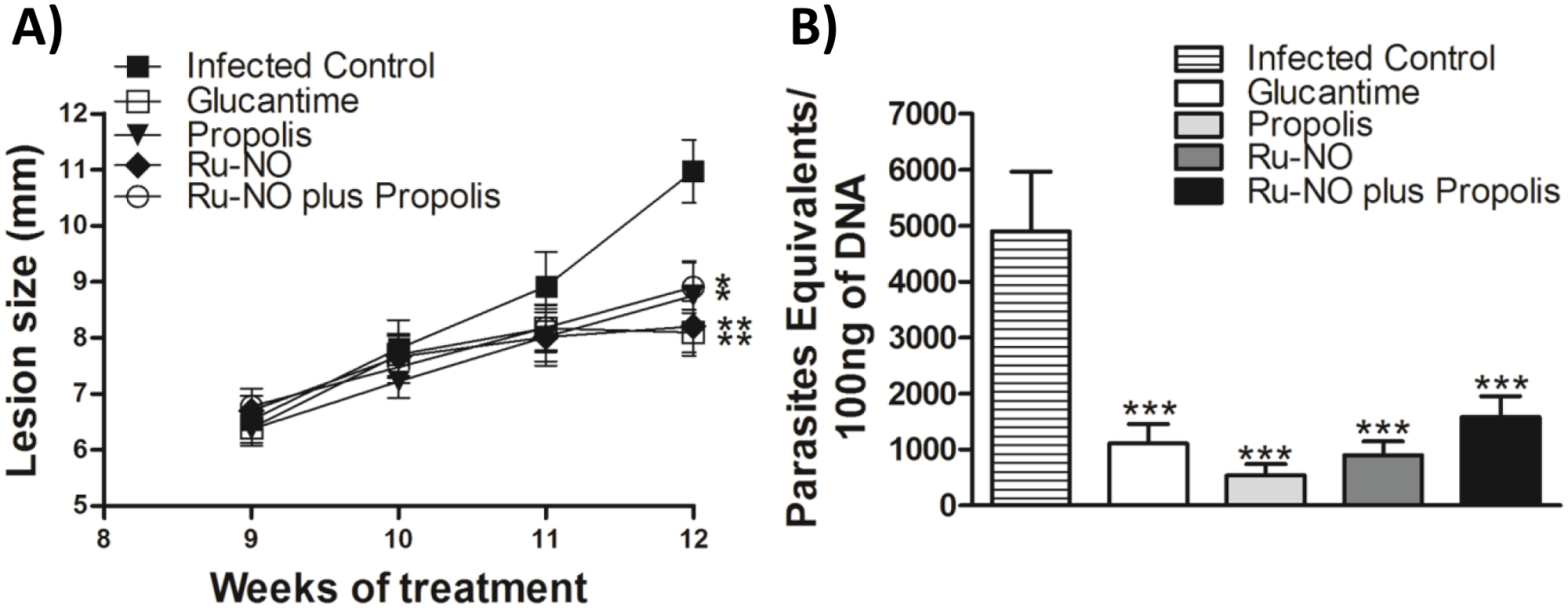
Effect of Ru-NO and propolis treatment on lesion development and parasite load of BALB/c mice infected with *L*. *amazonensis*. Animals were inoculated in the right hind paw with 1 x 10^5^ promastigote forms. **A)** After eight weeks of infection, mice were treated with saline (i.p.) (■), Glucantime (33 μmol.kg^-1^ day^-1^, i.p.) (□), propolis (5 mg.kg^-1^ day^-1^, p.o.) (▼),Ru-NO (0.385 μmol.kg^-1^ day^-1^, i.p.) (◆) or Ru-NO plus propolis (0.385μmol.kg^-1^ day^-1^, i.p. + 5 mg.kg^-1^ day^-1^, p.o.) (◯) for 30 days, and the lesion was measured once a week. **B)** At the end of treatment, the number of *Leishmania* kDNA was determined by real-time quantitative PCR. The results represent the mean ± SEM of lesion size for each group (n = 5). Significant difference relative to the infected control * *P*<0.05 and ** *P*<0.01, unpaired t-test.

The lesions of the infected control group increased progressively, and the ulcer size ranged from 9.7 to 12.5 mm at day 30 of treatment (Fig 3A). In contrast, the lesions in all treated groups showed a significantly slower development compared to infected control. At day 30, mean lesion size was 8.75±1.32 mm in the propolis-treated group, 8.20±1.13 mm with RuNO, 8.91±1.03 mm with RuNO plus propolis and 8.10±1.01 mm with Glucantime (Fig 3A).

Concerning the efficacy of treatments on parasite load, we determined parasite DNA levels at the lesion site by real-time PCR. A marked reduction in *L*. *amazonensis* number at the lesion site was found in all treated groups with no difference between them (Fig 3B). Interestingly, treatment with Ru-NO, propolis or Ru-NO plus propolis produced similar results as the Glucantime group. Furthermore, cutaneous lesions and parasite load in all treated animals were smaller than those found in the untreated infected control, showing the establishment of an inflammatory process with reduced tissue parasitism and severity of lesions.

It was important to assess the possible toxicity of combined treatment, and we found that Ru-NO plus propolis treatment daily for 30 days did not foster hepatic lesions since the hepatic enzymes ALT and AST remained unchanged (S1 Fig).

### Ru-NO plus propolis treatment increased the infiltrating macrophage population and modulated the expression of inflammatory markers

The complex interactions between *Leishmania* and immune cells have fundamental effects on the outcome of the disease. The success or failure of infection depends on the development of adaptive immune response. To investigate which immune cells migrate to the site of lesion and their role in the modulation on the cytokines synthesis after the treatment with Ru-NO and propolis, we performed histopathological analysis of the cellular infiltrate by light microscopy, immunohistochemical analyses for inflammatory markers and Western blotting to determine the expression of cytokines and transcription factor.

**Fig 4:**
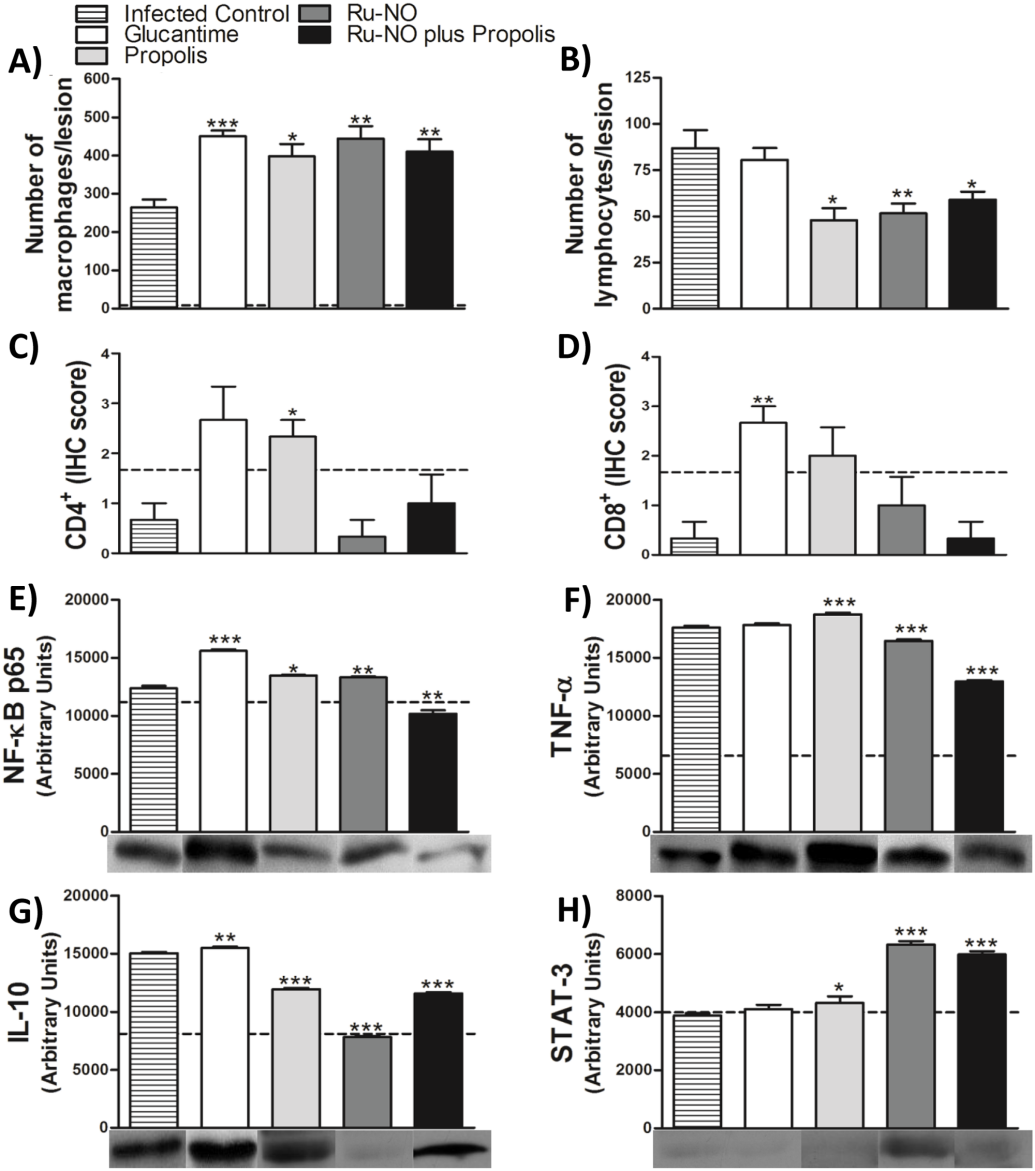
Effect of Ru-NO plus propolis on inflammatory process at lesion site. BALB/c mice were infected with 1x10^5^ promastigotes of *L*. *amazonensis* in the right hind paw and treated with Ru-NO (0.385 μmol.kg^-1^ day^-1^,i.p.), propolis (5 mg.kg^-1^ day^-1^, p.o.) or Glucantime (33 μmol.kg^-1^ day^-1^, i.p.) for 4 weeks post-lesion appearance. The paw sections were analyzed for number of **A)** macrophages (H&E), **B)** lymphocytes (H&E), **C)** CD4^+^ T (IHC), and **D)** CD8^+^ T (IHC) at a final magnification of 200x. The paw supernatants were assayed for expression of **E)** NF-κB(p65), **F)** TNF-α, **G)** IL-10 and **H)** STAT3 by Western blotting. The dotted line (—) represents the uninfected control. Bars represent the mean ± SEM (n = 5). Significant difference relative to the infected control * *P*<0.05, ** *P*<0.01 and ***P < 0.0001, unpaired t-test.

Regarding the cell profile of the lesion, the number of infiltrating macrophages was significantly increased at the lesion site in all treated groups when compared with infected control (Fig 4A). T cells were similar in the infected control and Glucantime-treated group. However, mice treated with propolis, Ru-NO or Ru-NO plus propolis exhibited a reduced T cell population (Fig 4B).

Immunohistochemical studies using specific monoclonal antibodies for CD4+ and CD8+ T labeling revealed that treatment with propolis and Glucantime increased the number of CD4^+^ and CD8^+^ T cells, respectively (Fig 4C and 4D). Nevertheless, Ru-NO only and the combined treatment Ru-NO plus propolis did not induce CD4 and CD8 recruitment to the lesion site (Fig 4C and 4D).

We evaluated the signaling pathways during the infection by measuring the expression of NF-κB (p65) (Fig 4E), TNF-α (Fig 4F), IL-10 (Fig 4G) and STAT3 (Fig 4H) by Western blot analysis of hind paw supernatant of each group. The expression of all markers was significantly altered during infection.

Glucantime treatment caused an increase in NF-κB and IL-10 expression compared to the infected control group. The animals treated with propolis showed a proinflammatory profile, with significant increase in expression of NF-κB, TNF-α and STAT3, associated with a reduced expression of IL-10, compared to infected control. The Ru-NO-treated group showed increased expression of NF-κB and STAT3 with concomitant reduction in the expression of TNF-α and IL-10 when compared to the infected control. Treatment with Ru-NO plus propolis caused a reduction in the expression of NF-κB, TNF-α and IL-10 and increased expression of STAT3, compared to the infected control. According to these findings, the combined use of Ru-NO and propolis seems to primarily impact the innate immune response, by improving the recruitment of effective macrophages to the lesion site, resulting in parasite killing besides the attenuation of the inflammatory process without any impact on the number of infiltrating T cells.

### Ru-NO plus propolis treatment did not promote the formation of NO-derived nitrotyrosine residues, favoring the control of lesion exacerbation

NO has been shown to be a crucial and versatile molecule in the control of a variety of intracellular organisms. In leishmaniasis, this compound is mainly produced by macrophages through the activation of inducible NO synthase (iNOS), which catalyzes the production of a huge amount of NO during infectious processes. Nevertheless, excess NO can yield peroxynitrite, which causes protein oxidation by nitration of tyrosine residues, resulting in nitrotyrosine formation and consequently increased tissue damage [45, 46]

**Fig 5:**
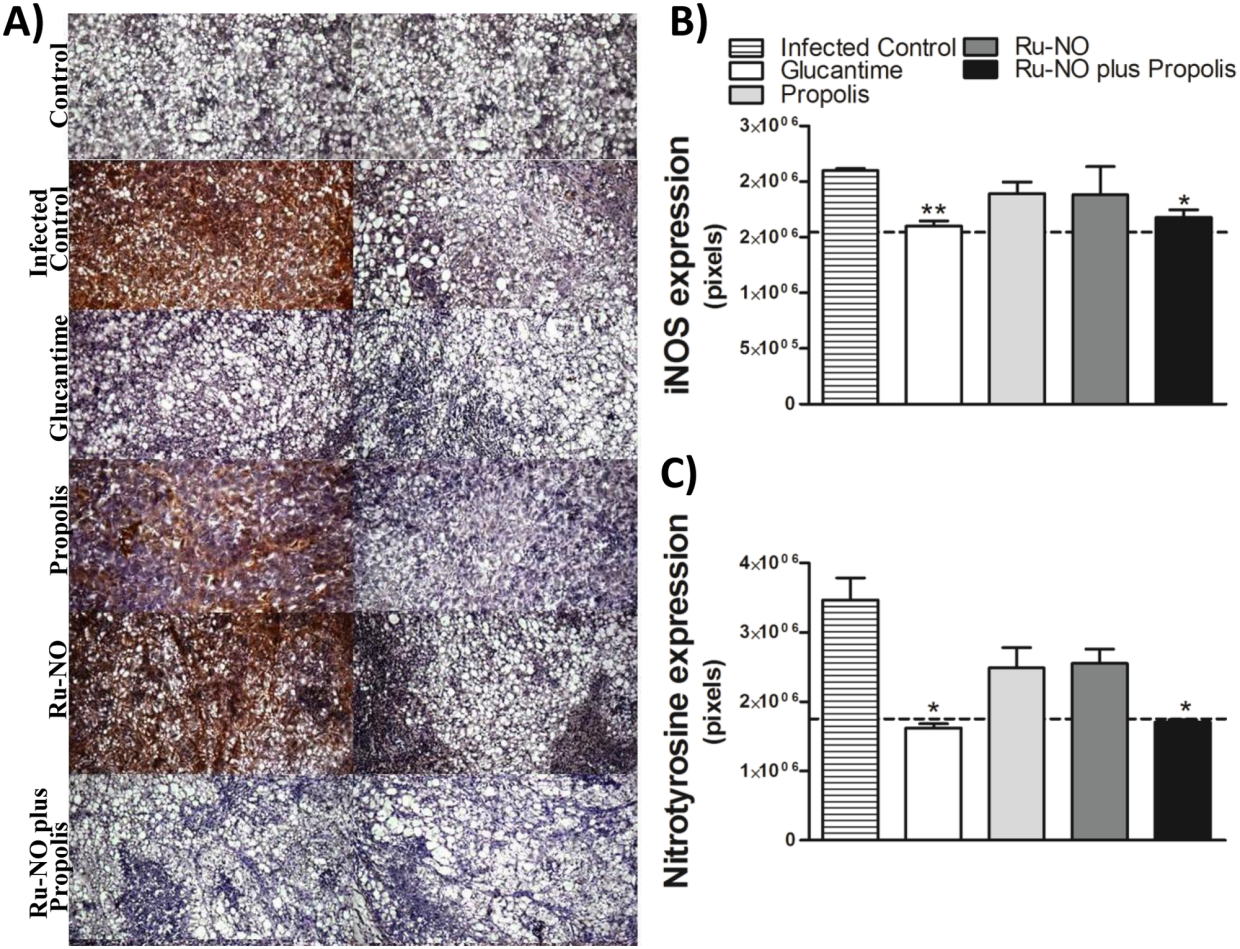
Ru-NO plus propolis decreased protein nitration at the lesion site. Expression of **A)** iNOS and **B)** Nitrotyrosine determined by immunohistochemical assays of paw sections of infected mice at 12 weeks post-infection. Infected BALB/c mice were treated with Ru-NO (0.385 μmol.kg-1 day-1,i.p.), propolis (5 mg.kg-1 day-1, p.o.) or Glucantime (33 μmol.kg-1 day-1, i.p.) for 4 weeks post-lesion appearance. The dotted line (—) represents the uninfected control. The result was expressed as the mean ± SEM of five animals per group. Significant difference relative to infected control * P<0.05, unpaired t-test.

To determine iNOS activity in treated groups and whether the administration of exogenous NO exacerbates tissue damage, we performed immunohistochemistry analysis. The results showed that after 30 days of treatment, the groups treated with Glucantime and with Ru-NO plus propolis showed lower iNOS expression when compared with the infected control group, returning to levels found in animals without infection (Fig 5A).

Concerning nitrotyrosine expression, the results were complementary and although all treated groups tended to show a decline in labeling, Glucantime and Ru-NO plus propolis groups showed similar nitrotyrosine levels as baseline levels also found in uninfected control (Fig 5B). Thus, the results indicated a reduction in the inflammatory process, demonstrating that exogenous NO supply along with the protective effects of propolis was beneficial to the host, favoring the control of the inflammatory response.

### Ru-NO plus propolis treatment induced tissue repair

Nitric oxide and propolis are also significant regulators of wound healing, and during healing of *Leishmania* lesions, there is a production of collagen and metalloproteinases [47]. We investigated the presence of tissue repair markers in the injured area.

**Fig 6:**
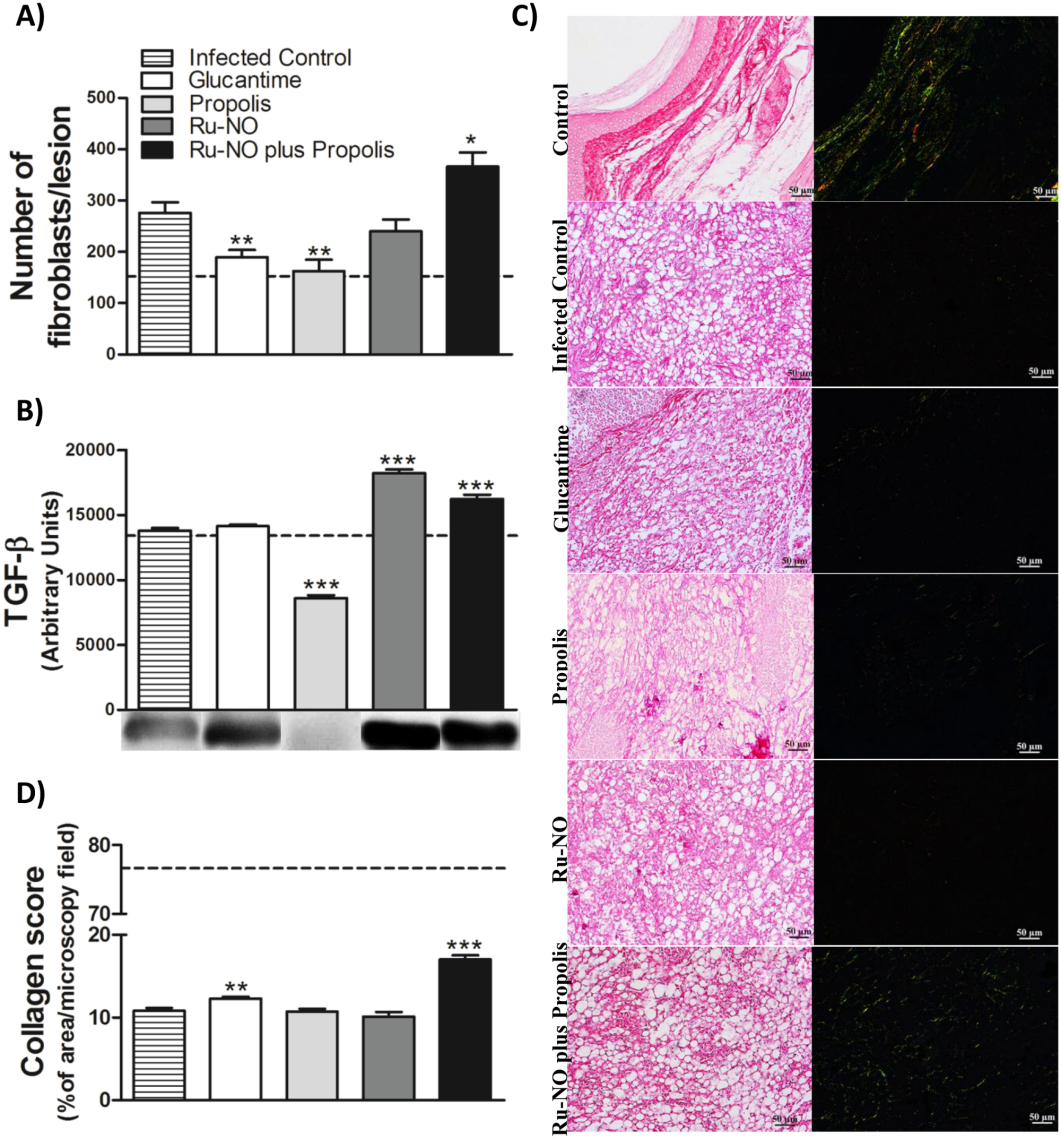
Effect of Ru-NO + propolis on healing process at the lesion site. The paw sections were analyzed for **A)** number of fibroblasts (H&E), **B)** TGF-β1 by Western blotting. **C)** Photomicrographs of paw sections stained with picrosirius technique **D)** and the quantification of collagen deposition. The paw sections were analyzed at a final magnification of 200x. The data represent the mean±SEM of five animals per group. The dotted line (—) represents the uninfected control. Significant difference relative to infected control ** *P*<0.01, ***P<0.0001, unpaired t-test. Scale bars = 50 μm.

Only the animals treated with Ru-NO plus propolis showed a considerable increase in fibroblasts at the lesion site, accompanied by the upregulation of TGF-β1 synthesis (Fig 6A and 6B). The groups that received propolis or Glucantime showed a decrease in fibroblasts in the lesions, and the Ru-NO group did not show any change in this cell type (Fig 6A). TGF-β 1 levels were also altered with a reduction in the propolis group and an increase in the group treated with Ru-NO only (Fig 6B).

Concerning the synthesis of collagen, picrosirius staining showed significantly greater levels of collagen deposition at the lesion site in the Ru-NO plus propolis group, indicating enhanced wound healing (Fig 6C and 6D). The group that received Glucantime also showed a slight increase in the amount of collagen fibers. However, in all other groups analyzed, collagen fibers were only weakly visualized at the injury site.

## Discussion

The inflammatory response developed during Leishmaniasis is essential for controlling the parasite burden, and the clinical outcome of ATL is a result of the immune capacity of the host in building an effective adaptive response against the parasite. In most cases, this host-parasite fight results in chronic disease and development of severe cutaneous lesions, where uncontrolled or continued inflammation is correlated with extended collateral damage and may constitute a crucial factor for the development of features of chronic disease (e.g., deforming ulcers) and subsequent morbidity [13, 14, 48].

In this context, the present study provided new perspectives for the treatment of experimental cutaneous leishmaniasis, especially regarding the use of combined therapy with the microbicidal molecule Ru-NO and the immunomodulatory propolis extract. Our data demonstrated that this combined therapy was able to increase the infiltration of competent macrophages at the site of leishmanial infection, decreasing the parasite load, modulating the inflammatory response and accelerating the healing of the lesion.

The analysis of the lesion development in infected footpad at 86 days p.i. showed that all treatments tested resulted in the control of the lesion, due to the enhanced recruitment of competent macrophages, consequently controlling the parasitic burden.

Several studies have shown that NO is involved in the death of *Leishmania* amastigotes during macrophage burst [7, 8, 49]. Recently, Olekhnovitch et al.[8] showed a new perspective of NO action in an experimental model of *Leishmania*. These authors proved that the collective production and subsequent diffusion of NO creates an antimicrobial environment that permits parasite killing in cells, demonstrating a cooperative mechanism at the tissue level to control *L*. *major* amastigotes, reinforcing our choice of an exogenous nitric oxide donor to control this disease.

Some molecules of a group commonly known as NO donors have been tested against *Leishmania* spp., showing a considerable effect against cutaneous leishmaniasis. The leishmanicidal effects of trans-[Ru(NO)(NH3)4L](X)3 have been demonstrated in *vitro* and *in vivo* against *L*. *major* [15]. *L*. *amazonensis*, when co-cultured with sodium nitroprusside *in vitro*, also showed a decrease in the number of promastigotes and axenic amastigotes in a concentration-dependent manner [50], and S-nitrosoglutathione (GSNO) was found to be cytotoxic to amastigotes and to promote the healing of topically treated *L*. *major* or *L*. *braziliensis* skin lesions[51].

Nevertheless, topical treatment of cutaneous leishmaniasis with NO donors, potassium nitrite and transdermal patches for continuous NO delivery, did not perform well against the disease [52, 53]. These variable results could possibly be related to the instability of NO release.

Our results demonstrated for the first time that the i.p. inoculation route of Ru-NO was effective in delivering NO to the lesion site, and consequently, the noted effect was due to the presence of NO and its reaction with compounds at the injury site and not to a systemic effect of this compound.

Regarding Brazilian propolis, our earlier study demonstrated that this propolis had antileishmanial and immunomodulatory effects against *L*. *braziliensis* in and *in vitro* experimental infection, increasing macrophage internalization and further killing of parasites [23]. The propolis tested contains mainly phenolic compounds, including flavonoids, di- and triterpenes, and essential oils [24]. In experimental leishmaniasis, propolis components such as prenylated compounds and benzophenones [54] have already been associated with the inhibition of amastigote proliferation by macrophage activation or by direct effect on promastigote forms (caffeic acid, p-coumaric acid, aromadendrine-4'-methyl ether, 3-prenyl-p-coumaric and 3,5-diprenyl-p-coumaric) [55]. Besides the leishmanicidal effects, peritoneal macrophages were activated by Brazilian green propolis, increasing the phagocytic ability [56].

When evaluating the combination of Ru-NO and propolis only with regard to amastigote number, we did not observe a greater reduction in parasite load; however, unlike with the other treatments, we found a considerable synergistic effect of these compounds in reducing the inflammatory process at the lesion site.

The effect of Ru-NO plus propolis on cell profile found in the lesions revealed a moderate inflammatory infiltrate, composed mainly of macrophage cells, and the quantification of CD4^+^ and CD8^+^ T lymphocytes showed a significant decrease in mice treated with Ru-NO plus propolis. It is possible that this attenuated inflammatory response had been, in part, due to reduced parasite load, suggesting that the combined drugs are crucial to promote parasite clearance and modulation of the local inflammatory response.

This immunosuppressive activity of NO has already been reported in *in vitro* and in *vivo* models, including microfilarie [57], mycobacterial [58] and *Toxoplasma gondii* [59] infections. NO donors are able to decrease the rolling and adhesion of leukocytes to endothelial cells, reducing transmigration to inflammatory sites [60–62].

Proving the alleviation of inflammation, we also observed reduced expression of TNF-α levels and transcription nuclear factor NF-κB, which induces several genes that are responsible for the coding of various proinflammatory mediators [63, 64], in mice treated with Ru-NO plus propolis. Propolis and Ru-NO, when administered alone, caused an increase in NF-κb transcription, and TNF-α level were also higher in the group treated with propolis.

Pavanelli *et al*. [65] also observed that treatment with the NO donor cis-[Ru(bpy)_2_(NO)SO_3_](PF_6_) decreased TNF-α production and consequently inflammation in an paracoccidioidomycosis infection model. The reduced production of TNF-α, IL-10 and INF-γ was also associated with reduced myocarditis in mice treated with the ruthenium NO donor trans-[RuCl([15]aneN_4_)NO]^2+^ in a Chaga’s infection model [66].

When evaluating the presence of protein tyrosine nitration, another marker of inflammation associated with the upregulation of iNOS [67], we observed that the expression of iNOS and nitrotyrosine levels were significantly diminished only in the groups treated with Ru-NO plus propolis and Glucantime, similar to that observed in the group without infection. Indeed, NO can control its own production by inhibiting iNOS activity, an important feedback control mechanism [68]. Interestingly, this result allows us to infer that while iNOS expression was low in the combined treatment group, NO delivered to the injury site did not aggravate the process of protein nitration, suggesting that this molecule may be consumed in another process. Taken together, the data suggest that the drug combination is crucial for parasite elimination and to modulate the local inflammatory response.

Another important property of both NO and propolis is the ability to promote tissue repair. In fact, in recent years, NO has emerged as a critical molecule in wound healing, where it increases collagen content in experimental wounds [69–71]. It should be noted that propolis extract is already used for wound healing, burns, external ulcers, shortening healing time, increasing wound contraction and accelerating tissue repair [72].

Soneja et al. [18] support the strategy of accelerating the wound healing process with antioxidants, leading to decreased oxidative stress and delivery of NO to the lesion site. The Ru-NO plus propolis treatment would be ideal for this approach, since various compounds in propolis have been described as powerful antioxidants, capable of scavenging free radicals, making the donated NO available for the healing process [73, 74].

Our results showed that only Ru-NO plus propolis treatment was able to induce the healing process, because it increased fibroblasts, a cell type that is a central player in tissue repair [17, 18], TGF-β and STAT3 levels and collagen deposition. IL-10 production was not altered by combined treatment, indicating that repair of tissue damage was not dependent on this cytokine.

TGF-β is a cytokine involved in orchestrating wound repair and participates in all phases of the healing process [75]. This cytokine helps in the chemotaxis of fibroblasts to the site of injury and stimulates collagen deposition [76, 77]. Nakamura et al. [78] and Frank et al. [17] showed that NO was able to convert latent TGF-β1 to the active form in fibroblasts, regulating collagen synthesis, where it is predominantly expressed during the repair process [79]. Transcription factor STAT3 when activated by various cytokines and growth factors plays a key role in wound healing [80]. Studies have shown that tissue-specific STAT3 gene deletion leads to impaired tissue remodeling [80–82].

Propolis, NO and TGF-β1 play central roles in collagen synthesis and can cross-regulate each other [33, 83, 84]. The synergistic signals of Ru-NO and propolis at the lesion site, provide a rapid deposition of remodeling tissue at the lesion site. Our data are in line with the findings of Baldwin et al. [85] in an ear model of *Leishmania* infection, which showed that the collagen deposition was more prominent in areas where re-epithelialization had occurred, when parasites had been cleared and inflammation controlled.

The data clearly showed that combined therapy resulted in alleviation of the inflammatory response, protected against tissue damage, and significantly accelerated the healing process when compared to the other groups.

It is important to emphasize that our therapy showed very similar effects as Glucantime conventional treatment, in relation to the control of lesion size and reduced amount of parasites. However, when considering the inflammatory response, which can be so aggravating in this parasitosis, and the induction of the healing process, there is no doubt that the effect of Ru-NO plus propolis was superior to that of Glucantime, also highlighting the lack of hepatotoxic damage in this treatment period.

In summary, the data obtained strongly suggest that the combination of *cis*-[Ru(NO)(bpy)2imN] (PF_6_)_3_ and Brazilian propolis is effective against *L*. *amazonensis in vivo*, allowing us to infer that this combined therapy can be an alternative for the treatment of leishmaniasis.

## Supporting Information

S1 Fig Effect of treatment with Ru-NO + propolis or saline on ALT and AST levels. BALB/c mice were treated with Ru-NO (0.385 μmol.kg^-1^ day^-1^, i.p.) and propolis (5 mg.kg^-1^, p.o.) for 30 consecutive days. ALT and AST were measured 24 h after the last treatment day (n = 5). Results are expressed as mean ± SEM * indicates *P* < 0.05 versus control, unpaired t-test.
(TIF)
