# Supplementary figures and images for: Nitric Oxide and Brazilian Propolis Combined Accelerates Tissue Repair by Modulating Cell Migration, Cytokine Production and Collagen Deposition in Experimental Leishmaniasis
Source: PLoS One. 2015 May 14;10(5):e0125101. doi: 10.1371/journal.pone.0125101 (PMC4431861; doi:10.1371/journal.pone.0125101)

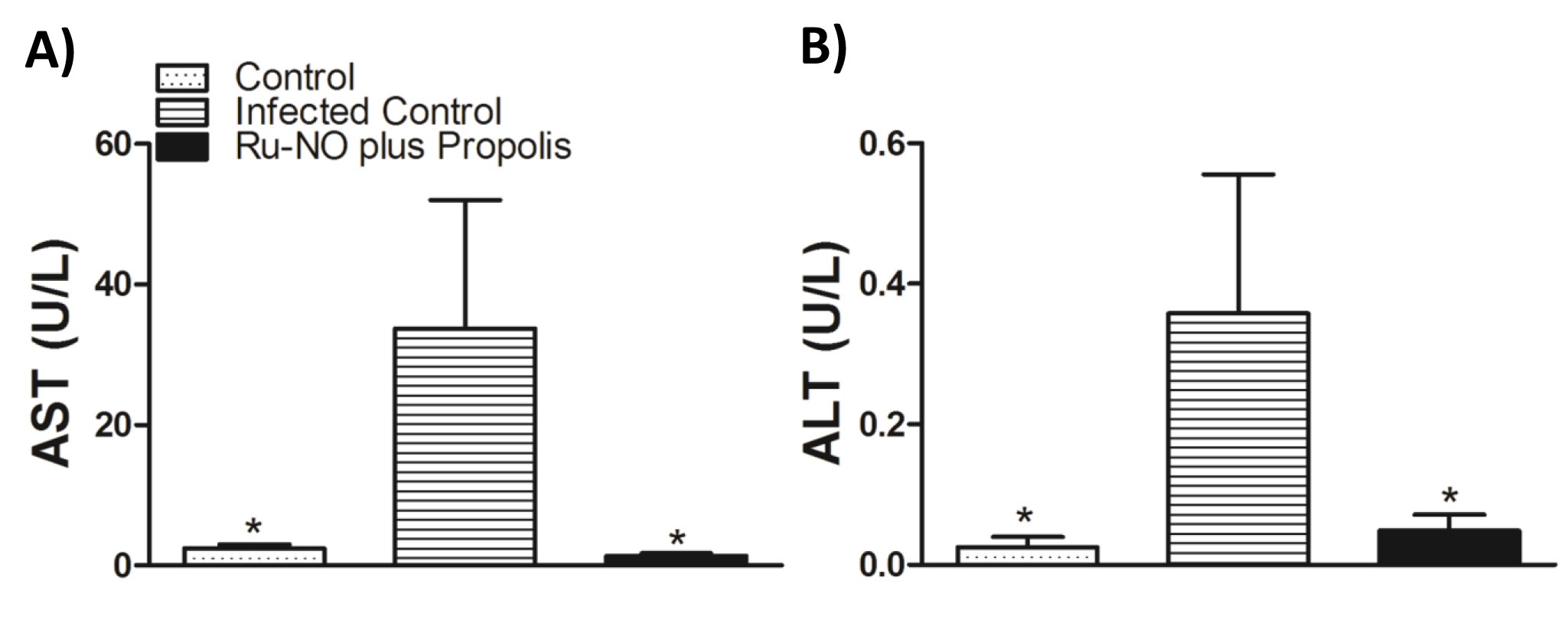

Supplement: S1 Fig — BALB/c mice were treated with Ru-NO (0.385 μmol.kg-1 day-1, i.p.) and propolis (5 mg.kg-1, p.o.) for 30 consecutive days. ALT and AST were measured 24 h after the last treatment day (n = 5). Results are expressed as mean ± SEM * indicates P < 0.05 versus control, unpaired t-test. (TIF) [file pone.0125101.s001.tif]
